# Nanocurcumin-Mediated Down-Regulation of Telomerase Via Stimulating TGFβ1 Signaling Pathway in Hepatocellular Carcinoma Cells

**DOI:** 10.22034/ibj.22.3.171

**Published:** 2018-05

**Authors:** Molood Shariati, Samira Hajigholami, Ziba Veisi Malekshahi, Maliheh Entezari, Narges Bodaghabadi, Majid Sadeghizadeh

**Affiliations:** 1 Department of Molecular Genetics, School of Biological Sciences, Tarbiat Modares University, P.O. Box 14115-154, Tehran, Iran; 2 Department of Medical Biotechnology, School of Advanced Technologies in Medicine, Tehran University of Medical Sciences, Tehran, Iran; 3 Department of Biology, Islamic Azad University, Tehran Medical Sciences Branch, Tehran, Iran

**Keywords:** Hepatocellular carcinoma, Telomerase, Gene expression

## Abstract

**Background:**

Curcumin, extracted from turmeric, represents enormous potential to serve as an anticancer agent. Telomerase is viewed as a prominent molecular target of curcumin, and transforming growth factor-β1 (TGFβ1) has proven to be a major inhibitory signaling pathway for telomerase activity. In the current study, we aimed to explore suppressive effects of nanocurcumin on telomerase expression through TGFβ1 pathway in a hepatocellular carcinoma cell line (Huh7).

**Methods:**

MTT assay was used to determine the effect of nonocurcumin on viability of Huh7 cells. RT-PCR was used to analyze the gene expression patterns.

**Results:**

MTT assay revealed that nanocurcumin acts in a dose- and time-dependent manner to diminish the cell viability. RT-PCR analysis indicated that nanocurcumin results in augmentation of TGFβ1 72 hours post treatment and leads to the reduction of telomerase expression 48 and 72 hours post exposure. Also, up-regulation of Smad3 and E2F1 and down-regulation of Smad7 confirmed the effect of nanocurcumin on intermediate components of TGFβ1 pathway. Furthermore, transfection of the proximal promoter of telomerase triggered a significant reduction in luciferase activity.

**Conclusion:**

The data from the present study lead us to develop a deeper understanding of the mechanisms underlying nanocurcumin-mediated regulation of telomerase expression, thereby presenting a new perspective to the landscape of using nanocurcumin as a cancer-oriented therapeutic agent.

## INTRODUCTION

Telomerase is a ribonucleoprotein complex with ability of synthesizing DNA at the end of chromosomes, playing pivotal roles in cell development, aging, and tumor genesis[1]. This enzyme comprises two constitutional subunits: an RNA component (hTER or hTERC) serving as a template for telomerase DNA synthesis and a catalytic protein (hTERT) with reverse transcriptase activity[2,3].

Telomerase function is essential for self-renewal and proliferation of some normal somatic cells, including stem cells, male germ cells, and activated lymphocytes, but its action is not detectable in most somatic tissues[1]. A plethora of studies have revealed that hTERT is permanently expressed in 70-90% of cancer cells functioning as a limiting factor for telomerase activity. Thus, mechanisms underlying the regulation of the hTERT gene are of great significance for cancer diagnosis and therapy[1-4]. The hTERT promoter harbors numerous binding sites for activating and inhibitory regulatory elements, including a variety of transcription factors, tumor suppressor, and oncogene proteins, hormones, and cytokines[5,6].

Transforming growth factor-β1 (TGFβ1) signaling pathway exists in species ranging from flies and worms to mammals. It has dual functions in tumor as suppression and promotion, depending on the tumor type and stage. It controls different cellular phenomena containing cell proliferation, recognition, differen-tiation, and apoptosis and notably functions as a potent tumor suppressor at the early stages of tumor genesis[7].

TGFβ1 signaling pathway has been shown to impede the hTERT gene expression and telomerase activity thorough its downstream factors in a diversity of cancers, such as kidney cancer, breast cancer, colorectal cancer, and lung cancer[8-10]. TGFβ1 is known to be as one of outstanding cytokines implicated in inhibiting the hTERT gene expression[8]. Gene expression can be controlled by Smad pathway, which is triggered by the binding of ligand to TGF-β receptors and consequently by the formation of Smad2/3/4 complex and translocation to the nucleus. TGF-β signaling can also activate MAPK pathways by applying other growth factors (non-Smad pathway)[11]. Upon activation through TGFβ1 receptors, R-Smads (receptor-regulated Smads) form a complex with co-mediator Smads, followed by translocation of the complex into the nucleus in order to regulate transcription of target genes. I-Smads (inhibitory Smads) can hamper this pathway via competition with R-Smads for binding to TGFβ1 receptors or recruitment of ubiquitin ligases to induce degradation of TGFβ1 receptors and other Smad proteins[12].

It has been demonstrated that TGFβ1 signaling pathway imposes its inhibitory action on the hTERT promoter by using several mechanisms. Studies have confirmed that TGFβ1-mediated repression of hTERT transcription is primarily triggered by Smad3 via direct binding to a specific motif on the hTERT promoter[9,10,13]. On the other hand, E2F1 has been proven to be a prominent mediator of TGFβ1 inhibitory effect on the hTERT gene expression[8,14]. Due to the documented role of telomerase in carcinogenesis, inhibition of the activity of this enzyme in cancer cells has received considerable attentions in cancer therapy[15-17].

Curcumin, a yellow-colored polyphenol pigment derived from the rhizome of the perennial herb *Curcuma longa* (well-known as turmeric), has long been used in Ayurveda medicines and has a diverse range of biological activities such as antiviral, anti-oxidant and anti-inflammatory properties[18,19].

Curcumin with the antioxidant and anti-inflammatory properties has been considered as a therapeutic agent in the control of liver cancer, which is highly affected by oxidative stress and inflammation condition[20]. Furthermore, within the past decades, a large body of evidence has underlined the therapeutic capability of curcumin versus cancer.[21-25]. In spite of the pharmacological safety and efficiency of curcumin as a potential agent for cancer treatment, its limited bioavailability, low solubility, poor pharmacokinetics, and low stability in aquatic environments has been highlighted as a serious obstacle for clinical applications[18,26]. To overcome this hurdle, various nano-formulations, including liposomes, micelles, adjuvants, and phospholipid complexes have been exploited to enhance serum half-life and tissue permeability of curcumin[26,27]. Our previous studies showed the capacity of PEGylated lipid-based nanocurcumin to inhibit the proliferation of cancer cells *in vitro* and *in vivo*[28-30].

In this study, we aimed to evaluate the suppressive effect of nano-formulation of curcumin developed in our laboratory on the hTERT gene expression via the induction of TGFβ1 signaling pathway in Huh7 cells, as a hepatocellular carcinoma cell line. The expression analysis of the genes belonging to TGFβ1 signaling pathway after treatment with nanocurcumin revealed that this agent can mediate the suppression of telomerase via triggering TGFβ1 pathway, representing the promise of this formulation of curcumin to be used against cancer cells.

## MATERIALS AND METHODS

### Cell culture

Huh7 cells (Pasteur Institute of Iran, Tehran) were cultivated in Dulbecco’s modified Eagle’s medium (DMEM; Gibco, USA) supplemented with 10% (v/v) fetal bovine serum (FBS; Gibco, USA) and antibiotics (100 U/ml penicillin and 100 U/ml streptomycin; Gibco, USA). Afterwards, the cells were incubated in a humidified atmosphere containing 5% CO_2_ at 37 °C for 24, 48, and 72 h.

### 3-(4,5-dimethythiazol- 2-yl)-2,5-diphenyltetrazolium bromide (MTT) assay

Nanocurcumin was prepared as previously reported[28]. The viability of Huh7 cells treated with nanocurcumin 24, 48, and 72 hours post exposure was evaluated thorough MTT assay according to the manufacturer’s protocol (Sigma-Aldrich, USA). Briefly, Huh7 cells were seeded in a 96-well plate in triplicate wells and grown in 200 μl of DMEM medium for 24 hours. Then the cells were exposed to a fresh medium containing concentration ranges (0-60 μM) of nanocurcumin for 24, 48, and 72 hours. With the completion of treatment period, 20 μl MTT solution (5 mg/ml in PBS) was added to each well, and the cells were incubated at 37 °C for 4 hours. Thereafter, the medium was removed, and the cells were lysed by adding 200 μl dimethylsolfoxide (DMSO) capable of dissolving formazan crystals produced by viable cells. The optical absorbance was measured at 540 nm using a 96-well plate ELISA reader (TECAN, Switzerland), and the effective concentration of nanocurcumin at which 50% of Huh7 cells were viable (IC50) was determined by the standard curve method[31]. Each experiment was repeated at least three times.

### RT-PCR

To analyze gene expression, Huh7 cells were seeded in six-well plates and cultured overnight. The cells were then treated with 20, 15, and 12.5 µM concentrations of nanocurcumin in triplicate wells for 24, 48, and 72 hours, respectively. For reverse transcription reactions, total RNA was isolated from treated cells, using Trizol reagent (Invitrogen, USA) according to the manufacturer’s protocol. cDNA was synthesized from 1 µg total RNA using PrimeScript™ 1^st^ strand cDNA Synthesis (Takara, Japan) with oligo dT (Invitrogen, USA). Primer sequences used for PCR reactions were as follows: hTERT (F: 5’-TTTGGTGG ATGATTTCTTGTTGG-3’; R: 5’-CACTGTCTTCCG CAAGTTC-3’), TGFβ1 (F: 5’-ACAATTCCTGGCGA TACCTC-3’; R: 5’- AGTGTGTTATCCCTGCTGTC-3’), Smad3 (F: 5’-GGAGGAGAAATGGTGCGAG AAG-3’; R: 5’- CACAGGCGGCAGTAGATGAC-3’), Smad7 (F: 5’-CGGAAGTCAAGAGGCTGTGT-3’; R: 5’-CATCGGGTATCTGGAGTAAGGAG-3’), E2F1 (F: 5’-AAGTCCAAGAACCACATCCAG-3’; R: 5’-TGCGTAGTACAGATATTCATCAGG-3’), GAPDH (F: 5’-GTGAACCATGAGAAGTATGACAAC-3’; R: 5’-CATGAGTCCTTCCACGATACC-3’). RT-PCR was carried out using Ampliqon Taq DNA Polymerase 2× Master Mix Red (Denmark) according to the manufacturer’s protocol with the optimized amount of cDNA for amplification of each gene in PCR reactions. The number of amplification cycles for any primer set was determined in order to be in the exponential phase. PCR products were subjected to electrophoresis on a 1.5% agarose gel and then visualized with ethidium bromide. The amplification products for each sample were normalized by using the *GAPDH* gene signal, and ImageJ software (version 1.51q) was then exploited to analyze the results of RT-PCR. Each experiment was performed at least three times.

### Recombinant plasmid construction and transfection

To create recombinant vector containing the hTERT promoter region (interacting with TGFβ1 signaling pathway)[8,9,32], PCR was performed (Ampliqon Taq DNA Polymerase 2× Master mix Red, Denmark) on blood-extracted genomic DNA as template using forward 5’-CGG**GGTACC**CCGCAGCTGCGCTGTC-3’ and reverse 5’-CCC**AAGCTT**GGGCAGCGCTGCC TG-3’ primers, including *Kpn* I and *Hind* III restriction sites (bold sequences), respectively. The digested fragments with *Kpn* I and *Hind* III restriction enzymes were gel purified (GeneAll^®^ Expin™ Combo GP, Korea) and then inserted into the *Kpn* I and *Hind* III sites of the digested promoterless PGL4.14 plasmid (Promega, USA) by T4 DNA ligase. The cloning procedure was confirmed by colony PCR and sequencing. For transfection, Huh7 cells (~6 × 10^4^ cells per well) were seeded in 24-well plates in triplicate 24 hours prior to experiment. Then 1 μg recombinant PGL4.14 plasmid with 2 µl lipofectamine 2000 were transfected into Huh7 cells using lipofectamine 2000 according to the manufacturer’s instruction (Invitrogen, USA), and cells were then maintained in an incubator with 5% CO_2_ at 37 °C for 6 hours. After this incubation, the media were replaced with a fresh DMEM containing 10% fetal calf serum and 1% penicillin-streptomycin, and the plates were incubated in an incubator with 5% CO_2_ at 37 °C for 24 h. Transfected cells were then treated with 20 µM concentration of nanocurcumin and incubated for an additional 24 hours. Afterwards, the cells were harvested in the cold CCLR lysis buffer, and then 10 μl cell lysate was employed to assay luciferase activity by luminometer (Berthold Detection Systems GmbH, Germany). All experiments were repeated at least three times[33].

### Statistical analysis

All experiments were analyzed by one-way ANOVA and Student’s *t*-test using GraphPad Prism 5. Data were presented as mean ± SD and for statistically significant differences. A value of *p* < 0.05 was considered statistically significant.

## RESULTS

### The effects of polymeric nanocurcumin on the viability of Huh7 cells

**Fig. 1:**
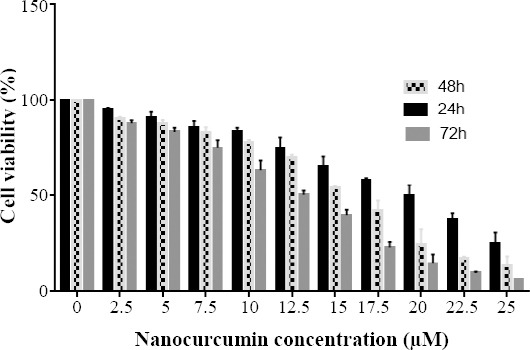
MTT assay showing the effect of nanocurcumin on the viability of Huh7 cells in a dose- and time-dependent manner. IC50 of nanocurcumin-treated Huh7 cells was determined as 20, 15, and 12.5 µM for 24, 48, and 72 h post exposure, respectively.

The safety of nanocarriers has been previously evaluated on various cancer cell lines in our laboratory[24-26]. MTT assay was conducted to investigate the viability of Huh7 cells 24, 48, and 72 h following treatment with various concentrations (0-60 μM) of nanocurcumin. The concentration of nanocurcumin in which 50% of Huh7 cells survived was determined as 20, 15, and 12.5 µM for 24, 48, and 72 h post exposure, respectively (Fig. 1). The best results were obtained from 72 hours experiments with 12.5 µM IC50. The data obtained thorough MTT assay revealed that nanocurcumin acts in a dose- and time-dependent manner on the viability of Huh7 cells because dose deduction of nanocurcumin is significant, in both times of 48 and 72 (*p* < 0.0001). This result leads us to the speculation that the increase of cellular exposure time as well as concentration of nanocurcumin significantly diminishes the growth and survival rate of Huh7 cells.

### Nanocurcumin-mediated suppression of hTERT expression through TGFβ1 family members

**Fig. 2:**
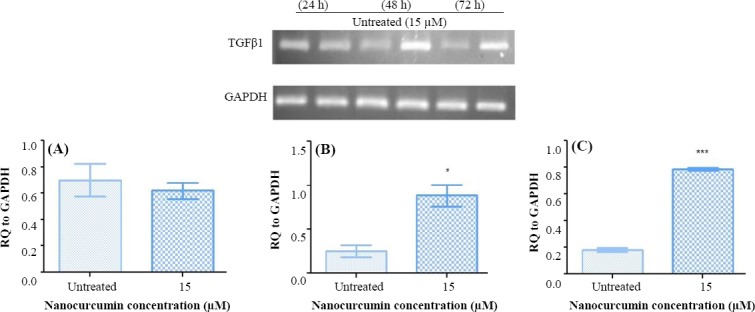
**Semi-quantitative** PCR analysis of TGFβ1 expression in Huh7 cells. The expression analysis of TGFβ1 in Huh7 cells post treatment with nanocurcumin indicated no significant alteration after (A) 24 h (*p* > 0.05), but a significant elevation was observed following (B) 48 and (C) 72 h (^*^p < 0.05 and ^***^p < 0.001, respectively).

**Fig. 3:**
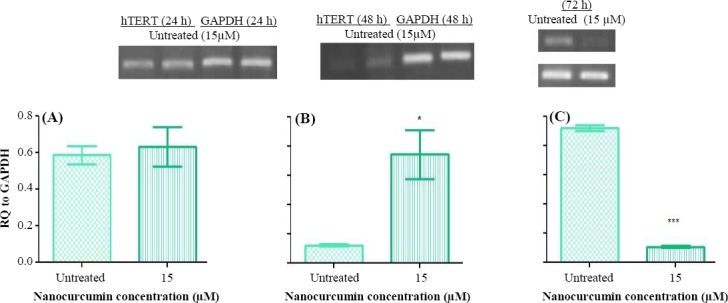
**Semi-quantitative** PCR RT.PCR analysis of hTERT expression in Huh7 cells. The expression analysis of hTERT in Huh7 cells post treatment with nanocurcumin showed an increase after (B) 48 h (^*^p < 0.05) and a decrease following (C) 72 h (^***^p < 0.001). There was not detected any significant alteration (A) 24 h post exposure.

**Fig. 4:**
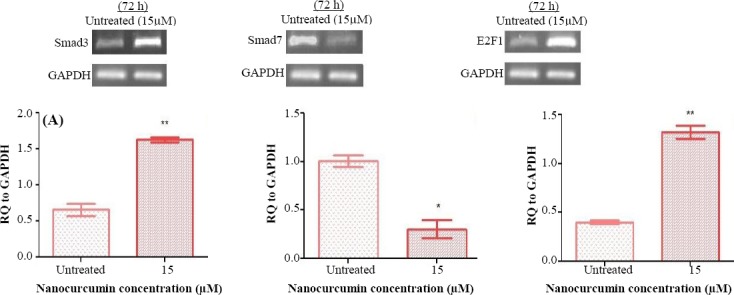
The results of the expression analysis of downstream effectors of TGFβ1 signaling pathway in Huh7 cells 72 h post treatment with nanocurcumin. The Figure illustrates the elevation of (A) Smad3 and (C) E2F1 expression (^**^p < 0.01) and the reduction of (B) Smad7 expression (^*^p < 0.05).

To study the suppressive effect of nanocurcumin on the hTERT gene expression through induction of TGFβ1 pathway, several doses of nanocurcumin were examined for gene expression analysis (data not shown). The results of the expression analysis of TGFβ1 and hTERT exhibited that there is not any significant alteration following 24 hours (*p* > 0.05), whereas a significant enhancement of expression occurs 48 h post exposure (*p* < 0.05; Figs. 2 and 3). Also, 72-h exposure of Huh7 cells to nanocurcumin led to the elevation of TGFβ1 expression (*p* < 0.001) and reduction of hTERT expression (*p* < 0.001; Figs. 2 and 3). Since inhibitory effect of nanocurcumin on the hTERT gene was observed 72 h post exposure, this incubation time was selected for analyzing the expression of TGFβ1 pathway members (Smad3 and Smad7) and E2F1 as a mediator of TGFβ1 with inhibitory function on hTERT. The results demonstrated a significant augmentation in Smad3 and E2F1 (*p* < 0.01), fundamental mediators of TGFβ1 with inhibitory function on hTERT, and a decline in Smad7 (principal suppressor of TGFβ1 signaling pathway) gene expression (*p* < 0.05) 72 h following treatment with nanocurcumin (Fig. 4).

### Nanocurcumin-induced inhibitory role of TGFβ pathway on telomerase by luciferase assay

**Fig. 5:**
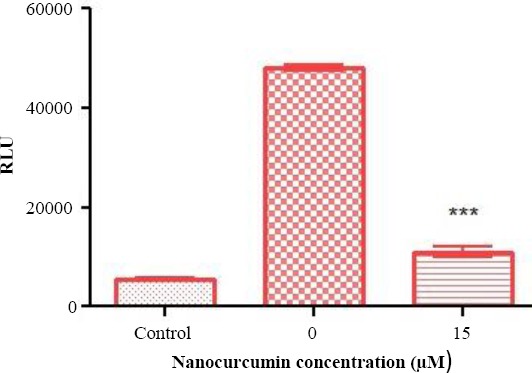
Luciferase assay through transfection of Huh7 cells with recombinant PGL4.14 plasmid harboring a region of the hTERT promoter. This region contains sequences previously shown to be able to interact with downstream mediators of TGFβ1 pathway. Transfection of Huh7 cells with the recombinant plasmid caused a significant reduction in luciferase activity (^***^p < 0.001) 24 h post exposure to 20 µM concentration of nanocurcumin. Relative light unit was used as the unit to quantitatively measure the expression level of lucifearse reporter gene. Promoterless PGL4.14 vector was employed as control.

Previous reports on the hTERT promoter have revealed the presence of a 255 to 300 bp region, proximal to start site, which is involved in TGFβ-mediated inhibition of telomerase activity[9,10,28]. To investigate the effect of nanocurcumin on the activity of hTERT promoter, the relevant promoter region was cloned into PGL4.14 (a promoterless vector containing luciferase reporter gene). The resulting construct was transfected into Huh7 cells, and the cells were then exposed to 20 μM concentration of nanocurcumin 24 h post transfection. Measurement of luciferase expression was indicated as relative light unit. The results of the transfection assay revealed that the exposure of Huh7 cells to nanocurcumin triggers a significant reduction in luciferase activity (*p* < 0.001) 24 h post treatment (Fig. 5).

## DISCUSSION

Curcumin, a natural herbal product, has received attentions as a potential agent in cancer therapy. This therapeutic capacity is rooted in anticancer properties of curcumin through which affect numerous molecular targets in malignant cells[34-36]. Telomerase is viewed as a prominent molecular target of curcumin in cancer cells[23-25], which plays critical roles in cell immortalization and carcinogenesis thorough the maintenance of chromosome ends[1,37]. Meanwhile, TGFβ1 is considered as a crucial effector in a signaling pathway known to be implicated in blocking telomerase activity[8,9,32,38]. Therefore, exploring the suppressive effects of curcumin on the hTERT gene expression by constituents of TGFβ1 pathway is of particular importance for translation into the clinic.

Recently, it has been noted that TGFβ1 signaling pathway mediates fibrogenesis in chronic diseases of the liver, kidney, lung, heart, and skin through overproduction and deposition of extracellular matrix components. Consistently, numerous studies have spotlighted anti-fibrogenic properties of curcumin by down-regulation of TGFβ1/Smad3 pathway[39-43].

Previous studies have underlined that the concentration of curcumin affects its antifibrogenic as well as anticarcinogenic properties. Curcumin exerts antifibrogenic effects at low concentrations (10 ≤ μM)[44], whereas it triggers apoptosis in cancer cells at high doses (10 ≥ μM)[45,46]. In spite of the recognized anti-fibrogenic role of curcumin via the inhibition of TGFβ1 pathway, to the best of our knowledge, there is not any report in the literature undertaken to study anticarcinogenic features of curcumin through induction of TGFβ1 pathway, as a potent inhibitor of the hTERT gene expression[47,48].

The current work reports on the augmentation of TGFβ1 expression and reduction of hTERT expression in nanocurcumin-treated Huh7 cells 72 h post exposure, implying the fact that nanocurcumin can lower telomerase expression via stimulating TGFβ1 signaling pathway in Huh7 cells. Expression analysis of the hTERT gene demonstrated an expression increase and decrease 48 h and 72 h post exposure, respectively. It has been confirmed that anti-neoplastic agents can generate genomic lesions and oxidative stresses provoking apoptosis in cancer cells[45,49]. However, dose and time exposure play essential roles in the response of cancer cells to anti-cancer compounds. Interestingly, a number of investigations have exhibited that genotoxic stresses prompted at

long-time low-dose exposure of cancer cells to anti-neoplastic agents could enhance the expression level of the hTERT mRNA. The elevated levels of the hTERT mRNA and subsequent increase of telomerase activity may be correlated with a survival advantage for cancer cells[50-53]. Consistently, our data suggest that up-regulation of the hTERT mRNA levels 48 h after treatment with nanocurcumin, as an anti-neoplastic compound[45,49,54], could raise the survivability of Huh7 cells.

Recently, it has been well documented that the rise of reactive oxygen species (ROS) production by curcumin suppresses telomerase activity, which in turn triggers apoptosis in cancer cell lines[1]. Therefore, we develop the hypothesis that nanocurcumin-driven enhancement of oxidative stress and ROS formation in Huh7 cells 72 h post exposure seems to disrupt the initial resistance of hepatocellular carcinoma cells and ultimately leads to the repression of the hTERT expression. Concentrations of more than 10 µmol of nanocurcumin have oxidant properties but concentrations of less than this amount of nano- curcumin have antioxidant properties[55]. Therefore, longer period of exposure can increase of the concentration of nanocurcumin inside the cell and generates more ROS. These contradictory alterations in the hTERT gene expression upon nanocurcumin-triggered genotoxic stress are presumably due to the fact that telomerase activation depends on the dose and duration of treatment. Various factors have been revealed to act at downstream of TGFβ1 to regulate telomerase activity. However, Smad3 (an R-Smad) and E2F1 are highlighted as the pivotal downstream mediators of TGFβ1 signaling pathway for preventing the hTERT expression[8,9,14]. Smad7 (an I-Smad) has been recognized to function as an inhibitory factor for Smad3 and negatively control the pathway[12]. The present study shows that nanocurcumin leads to the up-regulation of Smad3 and E2F1 and also down-regulation of Smad7. This finding reinforces the notion that nanocurcumin affects different mediators of TGFβ1 pathway ultimately resulting in hTERT repression. On the other hand, previous investigations have indicated the presence of a 255- to 300-bp region within the hTERT promoter, conferring TGFβ1 pathway prohibitive function on the hTERT gene expression[8,9,32].

To reveal an answer to the question that whether nanocurcumin can induce regulatory effects on the hTERT promoter through the induction of TGFβ1 signaling pathway, we constructed a recombinant PGL4.14 vector containing the relevant hTERT promoter region and transfected the resultant construct into Huh7 cells. The results suggested a significant suppression of luciferase activity post treatment with curcumin. This observation provides an insight into the inhibitory mechanism of nanocurcumin on the hTERT promoter through stimulation of TGFβ1 signaling pathway. It should be also noted that the precise mechanisms by which nanocurcumin impedes telomerase activity in a TGFβ1 signaling pathway-dependent manner remain to be clarified and necessitate further studies to decipher the interplays of nanocurcumin and TGFβ1 intracellular pathway implicated in inhibiting the hTERT gene expression. However, these data could shed light on the capability of nanocurcumin for TGFβ1 pathway-mediated regulation of the hTERT gene, thus representing potential of this nanoformulation of curcumin in the development of a novel approach for cancer therapy.
